# SRMR for Models with Covariates

**DOI:** 10.1017/psy.2024.10

**Published:** 2025-01-03

**Authors:** Daniel McNeish, Tyler H. Matta

**Affiliations:** 1 Arizona State University; 2 HMH

**Keywords:** Approximate fit, Conditional models, Covariates, Latent curve model, Latent growth model, Latent variable model, Model fit

## Abstract

The standardized root mean squared residual (SRMR) is commonly reported to evaluate approximate fit of latent variable models. As traditionally defined, SRMR summarizes the discrepancy between observed covariance elements and implied covariance elements. However, current applications of latent variable models often include additional features like overidentified mean structures and covariates, to which the traditional SRMR definition is not applicable. To date, SRMR extensions for models with covariates have received limited attention. Nonetheless, mainstream software provide SRMR for models with covariates, but values differ based on model specification and differ across programs. The goal of this paper is to formalize SRMR definitions for models with covariates. We develop possible SRMR definitions corresponding to different model specifications with covariates, discussing the advantages and disadvantages of each. Importantly, some SRMR definitions are susceptible to confounding misfit and model size such that SRMR values systematically decrease and suggest better fit when covariates are present, even if covariates have null effects. The primary conclusion is that there may not be a single unifying SRMR definition for covariates, but practically, researchers reporting SRMR with covariates should be aware (a) which definition is being used and (b) which information is and is not included in the particular definition.

## 1 Introduction

The standardized root mean squared residual (SRMR) has been characterized as a standardized effect size for evaluating the discrepancy between a model-implied covariance matrix and the covariance matrix from the observed data in structural equation models (Maydeu-Olivares, 2017; Maydeu-Olivares et al., 2018; Saris et al., 2009). Several recent sources have endorsed SRMR over competing fit indices like RMSEA or CFI based on advantages like a consistent interpretation that is less dependent on model characteristics (Shi et al., 2018; Ximénez et al., 2022), strong performance with small samples or small degrees of freedom (Pavlov et al., 2021; Shi et al., 2022), and the ability to put an interval around the index to account for sampling variability (Maydeu-Olivares et al., 2018; Ogasawara, 2001; Shi et al., 2020). SRMR also tends to be the least redundant with other commonly reported metrics (Hu & Bentler, 1998; Browne et al., 2002), and commonly cited resources for model fit evaluation have suggested a “two-index strategy” of reporting SRMR in conjunction with another index like RMSEA, CFI, Gamma Hat, or McDonald’s Centrality Index to minimize classification error rates (Hu & Bentler, 1999).

Although recent and classical research has extolled several benefits of SRMR, a potential limitation is that SRMR has not been rigorously studied—or formally defined—for some common types of structural equation models. The original definition of SRMR is valid for factor analyses where the mean structure is saturated or absent and where no covariates are present (Jöreskog & Sörbom, 1981; Bentler, 1995); however, the classical version of SRMR is not suitable for models that are interested in aspects beyond the covariance structure. For instance, mean structure models are the norm in most current applications because accommodating common missing data techniques requires a mean structure (e.g., Enders, 2006, p. 329), the fit of which may not be perfect even if the mean structure is saturated (Asparouhov & Muthén, 2018, p. 6). The traditional SRMR definition is insensitive to potential mean structure misfits and only incorporates covariance structure misfit (e.g., Leite & Stapleton, 2011; Wu & West, 2010).

Previous work has extended definitions of SRMR to include mean structures such that discrepancies between the observed and model-implied means can be incorporated into the index (e.g., Asparouhov & Muthén, 2018). However, other model features have not received much attention. In particular, covariates are present in latent growth models, multiple indicator multiple cause models (MIMIC), and some measurement invariance models but there is little formal study of the potential implications of covariates on SRMR definitions. Furthermore, covariates pose unique challenges related to the specification of covariates (i.e., fixed versus stochastic) and which model-implied moments are used (i.e., marginal versus conditional on covariates; Vonesh et al., 1996). As will be discussed shortly, these decisions impact which variables count as part of “the model” and can alter the numerator and/or the denominator of the SRMR calculation. Practically, this is relevant because different covariate specifications corresponding to the same conceptual model can have different SRMR values and implications for data-model fit.

Despite limited formal examination of SRMR extensions for models that include covariates, latent variable model software like M*plus* and lavaan currently output SRMR for models with covariates. As discussed in this paper, SRMR values in software output (a) do not agree across programs, (b) employ different SRMR definitions depending on which options are selected, or (c) may attempt to correct out covariate information with varying success.

The intention of this paper is therefore to (a) highlight the complexities of defining SRMR with covariates, (b) consider different possible SRMR definitions when covariates are present, and (c) better understand the advantages and disadvantages of different definitions. The ultimate goal is to help researchers make more informed and more accurate decisions when using SRMR to evaluate the approximate fit of their models. This issue is particularly timely because software programs are currently providing users with SRMR values even though such values are not well understood or may not align with the user’s expectations.

To outline the structure of the paper, Section 2 provides a brief example to motivate the nature of the issue. Section 3 overviews SRMR for covariance structure models and discusses recent extensions to mean structures. Section 4 reviews structural equation models with covariates and factors that complicate extensions of SRMR to these models. Section 5 outlines different ways that SRMR can be defined with covariates and how different model specifications impact what is included in different SRMR definitions. Section 6 provides an empirical application of a latent growth model with covariates to highlight how different versions of SRMR behave. A small simulation also demonstrates that the patterns in the empirical example hold when the population model is known. Section 7 concludes with limitations and future directions.

## 2 Motivation

To motivate the nature of the problem, consider data generated in M*plus* Version 8.10 from the following unconditional linear growth model with four repeated measures,(1)

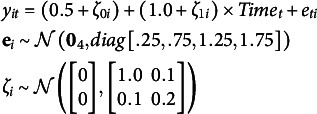

where 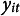 is the outcome from person *i* at time *t*, 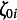 is a person-specific latent intercept, 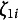 is a person-specific latent slope, and 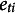 is within-person error for person *i* at time *t*. Each of the 500 simulated datasets, *i* = 1, …, 1000.

Five models are fit to each generated dataset using homoskedastic error variances to underparameterize the model so that fit is not perfect. The first model correctly specifies no covariates. The remaining four models add 1, 2, 3, or 4 time-invariant covariates as predictors of the latent intercept and slope, but each covariate is known to have no effect on the population. Because the null covariates do not explain any variance, the model with and without covariates is functionally the same and SRMR should seemingly not improve.

**Figure 1:**
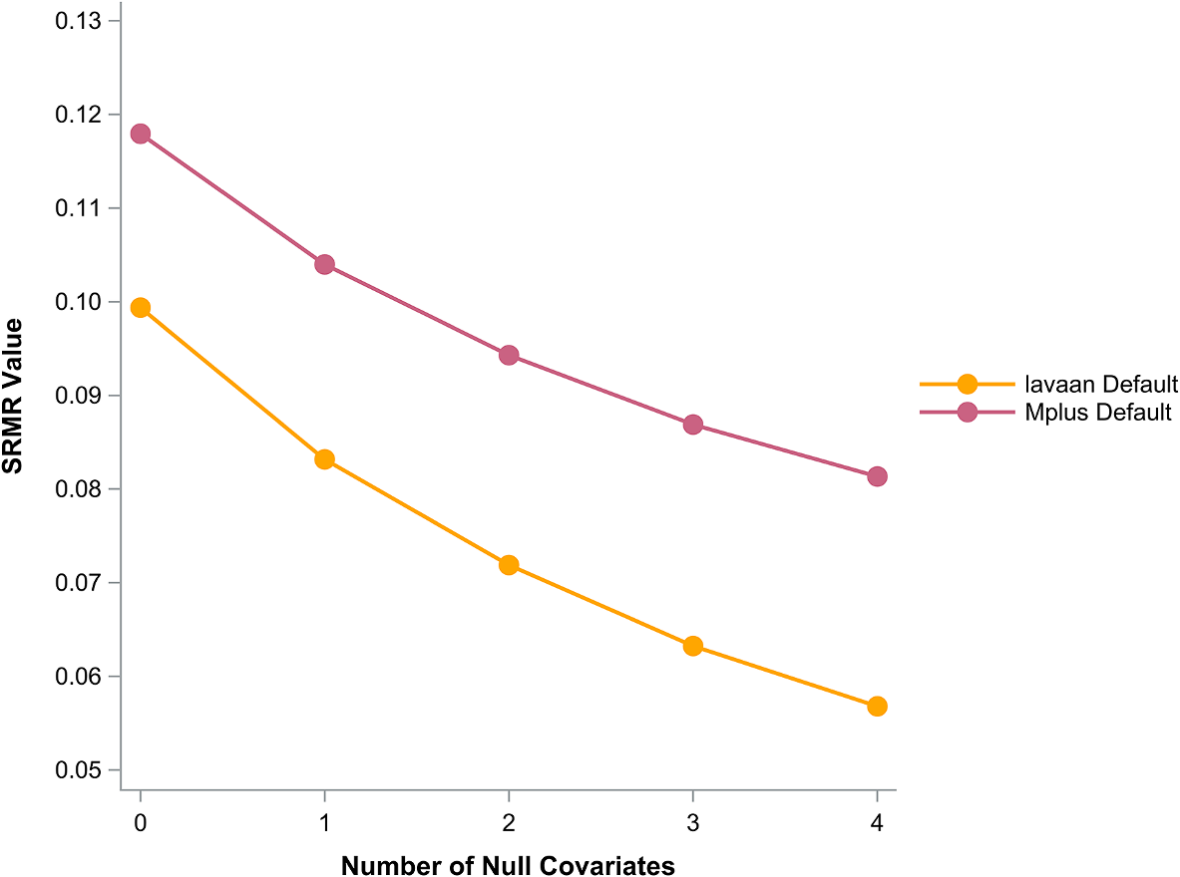
Average SRMR values across replications for a latent growth model with four repeated measures fit with default options in lavaan and M*plus*. The population model has no covariates, but null covariates were added. The SRMR value systematically decreases as a function of covariates, even though the covariates explain no variance and have no effect.

Figure 1 shows SRMR averaged across replications with default settings in M*plus* Version 8.10 (Muthén & Muthén, 1998–2017) and default settings in lavaan Version 0.6.17 (Rosseel, 2012). Importantly, SRMR values do not agree, and fit appears to steadily improve as more null covariates are added, counterintuitively suggesting better fit despite null covariates effects.

## 3 Overview of residual fit indices

### 3.1 Likelihood ratio test

Consider a population of random variables with mean 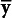 and population covariance 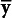 . A random sample of size *N* from this population has a data matrix **Y** with sample mean 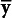 and sample covariance **S**. A structural equation model proposed to model relations between variables in **Y** has a model-implied mean structure 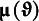 and a model-implied covariance structure 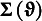 where 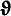 is the fundamental parameter vector containing the *f* freely estimated parameters featured in the model (Skrondal & Rabe-Hesketh, 2004). With maximum likelihood estimation, parameter estimates for 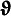 are found by minimizing the maximum likelihood discrepancy function,(2)

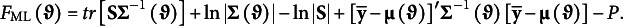



*P* corresponds to the number of variables in the model, dimensions of **S** and 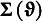 are each 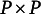 , and the dimensions of 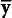 and 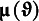 are each 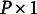 .

To test whether the model-implied moments exactly reproduce the sample moments, a likelihood ratio test statistic can be defined by 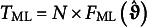 where *N* is the total sample size and 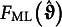 is the value of the discrepancy function evaluated at the maximum likelihood estimates of the parameters, 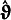 . Under the assumption of multivariate normality, *T*_ML_ is asymptotically distributed 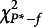 where 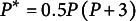 , the number of non-duplicated entries in the augmented covariance-mean matrix.

Though valued for its clear definition and inferential nature, researchers have noted that satisfying an exact fit test like *T*_ML_ is not always a necessary condition for a model to be useful (Bentler & Bonett, 1980; Hu et al., 1992; MacCallum, 2003). That is, models are often intended to be approximations from the onset, so tests of exact fit may be expected to be false a priori (e.g., Browne & Cudeck, 1993). Consequently, approximate fit indices like RMSEA, CFI, and SRMR have become popular supplemental metrics to summarize the practical magnitude of misspecifications throughout the model (Jöreskog & Sörbom, 1982).

Whereas *T*_ML_ is interested in the presence of misfit between the model-implied and observed moments, approximate fit indices are interested in quantifying the magnitude of the discrepancy between the model-implied and observed moments (e.g., McNeish & Wolf, 2023) and operate more like effect sizes for model misspecification (Kelly & Preacher, 2012). Commonly reported approximate fit indices like RMSEA and CFI are transformations of *T*_ML_, but SRMR is unique in that it is based on the model residuals (Yuan, 2005) where a “model residual” is the difference between a model-implied moment and an observed moment. SRMR can therefore have unique advantages relative to other indices and can provide non-redundant information. The remainder of this paper focuses on properties, clarifications, or extensions of SRMR.

### 3.2 SRMR for covariance structure models

Jöreskog and Sörbom (1982) first proposed the root mean residual (RMR) index based on the model residuals, which summarizes the difference between **S** and 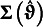 with a single value. RMR is unit dependent and can be unintuitive to interpret, so Bentler (1995) proposed the traditional classic definition of SRMR to standardize the RMR such that,(3)

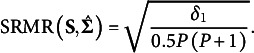

and(3a)

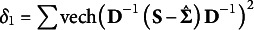


(3b)

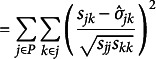


(3c)

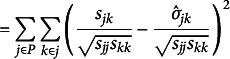


(3d)

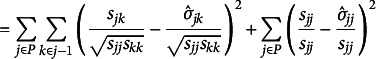


(3e)

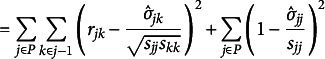



For 
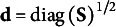
 , 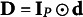 , 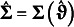 , and 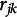 is a correlation element 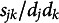 for 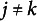 .

Equations 3b and 3c illustrate that the residuals, 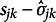 , are scaled according to the product of the *sample* variances ( 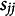 and 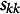 ) such that the denominator always consists of elements of **S**, even when the numerator is an element of 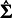 . Correspondingly, equation 3e shows that the minuend is a sample standardized metric (standardized covariance, 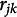 , or standardized variance, 1) because the numerator is divided by diagonal terms from the same matrix. However, subtrahend of equation 3e is the model-implied parameter estimate scaled by the product of the *j*th and *k*th *sample* standard deviation or the *j*th *sample* variance, respectively. Consequently, the model-implied elements are not necessarily completely standardized whenever the variances are not saturated because 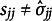 and 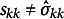 , which may occur in a growth model (e.g., if residual variances are constrained to equality across repeated measures).^1^ The denominator in equation 3 is 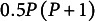 , which is the number of unique diagonal and off-diagonal elements of the covariance matrix.

The SRMR expressed in equation 3 only considers elements from the sample and model-implied covariance matrix but includes no information about the mean structure. It is, therefore, suitable for factor analysis where mean structures are absent or saturated, but not for models with overidentified mean structures like latent growth models (Leite & Stapleton, 2011; Wu & West, 2010). Structural equation model applications frequently feature an overidentified mean structure, so an extension of SRMR that incorporates the model residuals between the model-implied means and sample means (i.e., 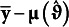 ) is desirable. Such an extension is described in the next section.

### 3.3 SRMR with a mean structure

Define 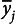 as the *j*th element of 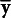 and 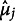 is the *j*th element 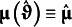 . The SRMR for a model with an overidentified mean structure therefore extends to(4)

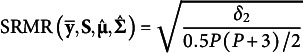

where(4a)

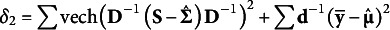


(4b)

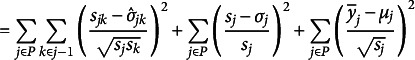


(4c)

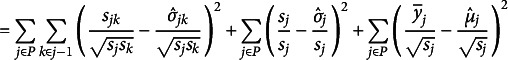


(4d)

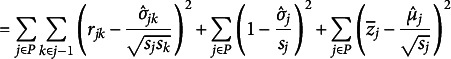



Importantly, equation 4a adds a new term 
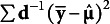
 to account for differences between the observed and model-implied means. The denominator in equation 4 changes to 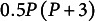 to incorporate elements in the mean structure. Like equation 3e, each residual in the minuend of equation 4d is a *sample* standardized metric (*r_jk_*, 1, or *z_j_*) while the subtrahend is the model-implied parameter estimate divided by the square-root of the product of the *j*th and *k*th *sample* variances, the *j*th sample variance, or the *j*th standard deviation, depending on the residual being standardized. In other words, the elements of 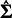 and 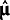 are divided by diagonal elements of **S**.

Note that the third term in equation 4d corresponding to mean structure residuals is unbounded. Conversely, the first term corresponding to the covariances is approximately standardized (depending on the congruence of diagonal elements in **S** and 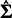 ) and will be bounded by a value near 2 (e.g., its maximum value occurs when the observed correlation is 1 and the model-implied correlation is –1). When a discrepancy between the covariance and mean structure is summarized by a single value, a large unbounded misfit in the mean structure may overpower the covariance structure misfit. Conversely, for large models, there can be many more covariance elements than mean elements and the covariance elements can wash out the contribution of the mean structure. It can therefore be prudent to separately examine the contribution of the covariance structure misfit and mean structure misfit (e.g., Yuan et al., 2019). The lavResiduals function in lavaan will provide separate SRMR values for all elements combined, only the covariance elements, and only the mean elements.

### 3.4 Alternative standardization methods

Whereas equations 3 and 4 standardize with sample standard deviations (sometimes called Bentler standardization), an alternative approach is to standardize model-implied moments by *model-implied* standard deviations rather than observed standard deviations (sometimes referred to as Bollen standardization; Bollen, 1989). With this standardization, the numerator in equation 3 would instead be 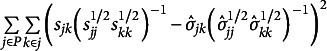 . This transforms the observed and implied covariance matrices to correlation matrices prior to taking the difference, which removes potential contributions of the diagonal terms because they will always be 1 in each matrix. Consequently, the index derived from this standardization is typically referred to as a separate index (the correlation root mean square residual; CRMR, Bollen, 1989) rather than SRMR.

There are also proposed definitions that mix Bollen standardization for the covariance and mean elements with Bentler standardization for the variance elements so they are not excluded (this definition is employed by default in M*plus*, Asparouhov & Muthén, 2018). Specifically,(5)

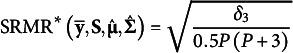

where(5a)

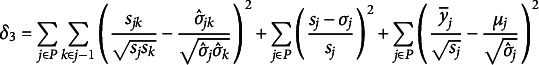


(5b)

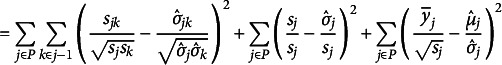


(5c)

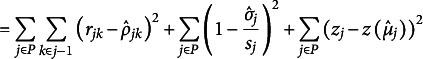



Notice that the first and third terms of equation 5a are divided by elements of 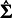 rather than **S** as in equations 3 and 4 but the middle term of equation 5a continues to divide by an element of **S**.

### 3.5 SRMR for models with covariates

As shown in equations 3–5, SRMR definitions heavily rely on the definition of *P*, which is prominently featured in the denominator of each definition. For models without covariates, *P* is unambiguous. However, when covariates are present, the situation becomes more opaque because covariates may or may not count as part of *P*. Additionally, models with covariates will have marginal and conditional structures depending on how a researcher wishes to treat the variance explained by covariates, which may complicate SRMR definitions.

Potential challenges of SRMR with covariates have been considered, but have yet to be more rigorously embraced. Section 4 overviews details and properties of models with covariates needed to discuss different possible SRMR definitions; definitions are then provided in Section 5.

## 4 Structural equation models with a mean structure and covariates

A general structural equation model with a mean structure and covariates can be written as,(6)

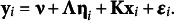



where 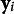 is a *P*-dimensional vector of manifest outcome variables for person *i* 
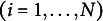
 , 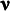 is a *P*-dimensional vector of manifest outcome intercepts, 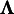 is a 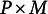 matrix of factor loadings for *M* the number of latent variables, 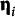 is an *M*-dimensional vector of latent variables, 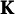 is a 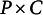 matrix of parameters associating the *C*-dimensional 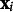 vector of manifest covariates for person *i* that directly predict to the manifest outcome 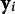 , and 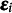 is a *P-*dimensional vector of residuals for person *i* such that 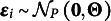 .

The structural model for the latent variables can then be written as(7)

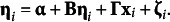



where 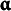 is an *M*-dimensional vector of latent variable means, **B** is an *M*-dimensional square matrix of structural paths between latent variables, 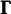 is an 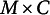 matrix of parameters associating the *C*-dimensional 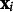 vector of manifest covariates for person *i* to the latent variables 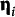 , and 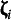 is an *M*-dimensional vector of disturbances for the latent variable for person *i* such that 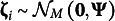 .

The fundamental parameters vector containing the unique parameters from equations 6 and 7 is 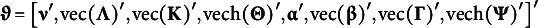 , which is featured in the estimator in equation 2 and is the basis for the model-implied means and covariances.

### 4.1 Model-implied means

From the estimated parameters in 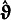 , the model-implied conditional expectation for the manifest outcomes in **y** given the covariates **x** can be expressed as(8)

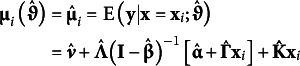



The *i* subscript on 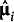 indicates the expectation changes as a function of covariate values.

When fitting a model conditional on the covariates, the resulting output is typically the expected values given 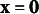 . This results in a different conditional expectation such that,(9)

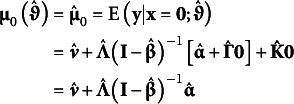



The “0” subscript denotes that the expectation is conditional on the covariate being equal to 0.

Setting the covariate values to their respective sample means, 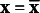 marginalizes over the covariates to arrive at a model-implied marginal expectation for the focal outcomes where(10)

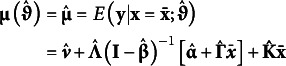


*i* subscripts are dropped in equation 10 to indicate a marginal expectation given that covariates are set to their respective sample means.

### 4.2 Model-implied covariance

The *P* × *P* model-implied covariance for the manifest outcomes, conditional on covariates, can be expressed as(11)

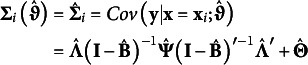



Like the model-implied conditional expectation, an *i* subscript indicates that the covariance is conditional. However, the conditional model-implied covariance does not vary as a function of covariate values (i.e., **x** does not appear in equation 11), so 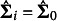 . Together, equations 8 and 11 define the conditional model-implied probability distribution such that 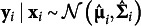 or 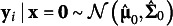 .

Correspondingly, the model-implied marginal covariance matrix is(12)

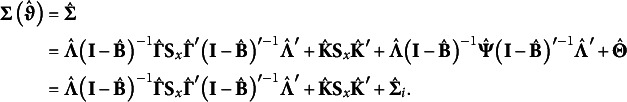



where 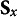 is the sample covariance matrix for the covariates. Notably, the model-implied marginal covariance is calculated from the conditional covariance matrix ( 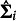 ) plus the proportion of variance in the outcomes that are explained through covariates. Together, equations 10 and 12 define the marginal model-implied probability distribution such that 
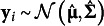
 .

### 4.3 Special case of continuous outcomes

When **y** is continuous and all covariates are exogenous, the model can be simplified based on LISREL notation. Namely,(13)

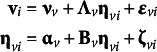

where 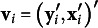 stacks all the variables into one vector and all variables are treated as outcomes. This notation does not permit direct paths from manifest variables to latent variables (e.g., manifest variables can only indicate latent variables, but they cannot predict them; Bollen 1989, pp. 395). Instead, single-indicator latent variables are created for each manifest variable that predicts or is predicted by another manifest variable where factor loadings are fixed to 1 and residual variances fixed to 0 for identification.^2^


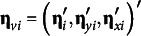
 is then composed of three parts, (a) focal latent variables ( 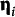 ), (b) dummy latent variables for elements of **y** that are predicted from elements of **x** ( 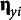 ), and (c) dummy latent variables for elements of **x** that predict elements of **y** or 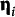 . All regression paths are housed in the **B** matrix rather than being split amongst **Γ**, **K**, and **B** as in equations 6 and 7 (Skrondal & Rabe-Hesketh, 2004, p. 78).

M*plus* and lavaan rely on this notation for efficient computation with continuous variables (Muthen, 2004, p. 13; von Oertzen & Brick, 2014). Other notation systems like reticular action model notation (RAM, McArdle & McDonald, 1984) or Bentler–Weeks notation (Bentler & Weeks, 1979) can directly accommodate paths from manifest covariates to latent variables. Correspondingly, different specifications emerge for models with covariates. Section 4.4 reviews these different specifications and Section 5 discusses implications for how different specifications can have different SRMR definitions.

### 4.4 Specifications for models with covariates

**Figure 2:**
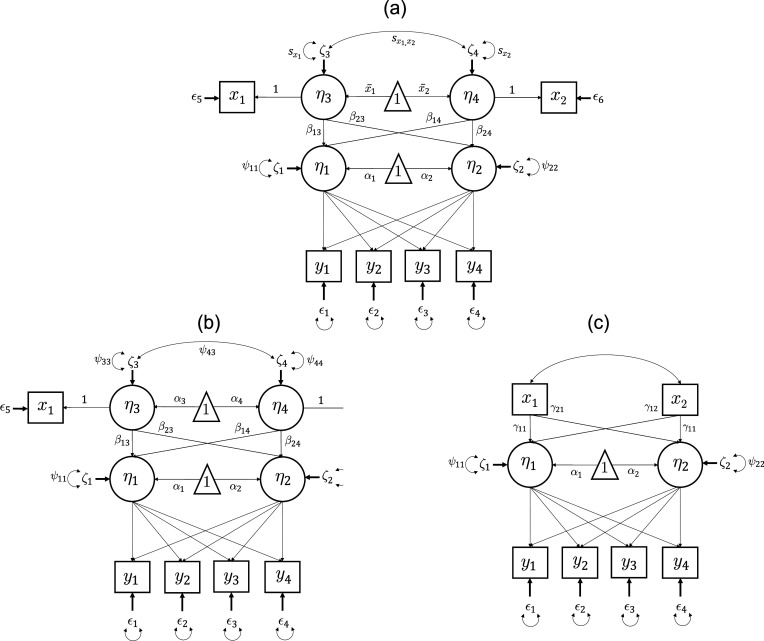
Hypothetical path diagram of conditional latent growth model with two time-invariant covariates and four repeated measures. Panel (a) shows a joint and fixed covariate specification where the covariates are converted to latent variables whose moments are constrained to sample statistics. Panel (b) shows a joint and stochastic specification where the covariates are converted to latent variables whose moments are free parameters. Panel (c) shows a conditional and fixed specification where the manifest covariates directly predict the latent growth factors. The difference between panels (a) and (b) is subtle and is related to whether the means, variances, and covariances of *η*_3_ and *η*_4_ are fixed or estimated.

There are two main dimensions along which model specifications with covariates can differ. The first is joint versus conditional, the second is fixed versus stochastic. The result is four possible combinations, though one combination (conditional and stochastic) is theoretically possible but seldom serviceable, so it is not considered here. Figure 2 illustrates the differences between model path diagrams for different specifications for a hypothetical conditional linear growth model with four repeated measures and two time-invariant covariates predicting the growth factors. Figure 2a shows the joint and fixed specification, Figure 2b shows the joint and stochastic specification, and Figure 2c shows the conditional and fixed specification. More details on each specification appear in dedicated subsections below.

#### 4.4.1 Joint and fixed

In the joint specification, a joint likelihood for the outcome variables and all covariates is built such that 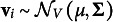 . In Figure 2a, this is represented by the manifest covariates *x*_1_ and *x*_2_ being replaced with single-indicator latent variable models with factor loadings fixed to 1 and their residual variances fixed to 0. These single-indicator latent variables then predict the latent growth factors. This specification is used by default in lavaan and M*plus* by default.

With a joint specification, all covariates become dependent variables in the model, which has ramifications for how *P* is defined within SRMR calculations. Because covariates technically become outcomes (i.e., a latent variable points into them), they are pulled into ‘the model’ such that *P* equals the sum of the *T* focal outcome variables in **y** and the *C* covariates in **x** predicting the latent variables or manifest outcomes. This sum is defined as *V* where *V* = *T* + *C.* The model-implied moments correspond to equations 10 and 12.

With a fixed specification, the mean, variances, and covariances of the covariates are constrained to their sample values rather than being estimated. Because there are no free parameters in the covariate portion of the model, full information maximum likelihood is not applicable with this specification and missing covariates must be imputed or listwise deleted.

The full model equations for the joint and conditional specification in Figure 2a are,(14)

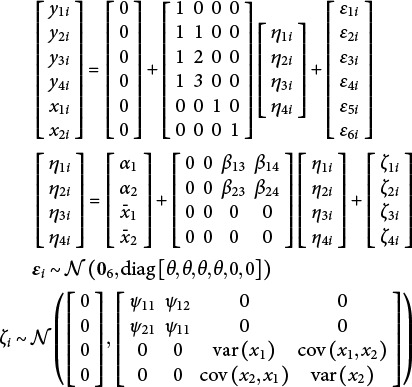



#### 4.4.2 Joint and stochastic

A joint and stochastic specification maintains the joint likelihood approach in Section Section 4.4.1 but differs in how covariates parameters are treated. Namely, rather than fixing the covariate means, variances, and covariances to their sample values, these parameters are directly estimated. That is, the vector of latent variables means in equation 14 would change to 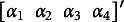 and the lower right triangle of the disturbance covariance matrix in equation 14 would change to 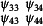 . This can be seen in Figure 2b where sample statistics 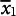 , 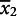 , 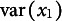 , 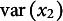 , and 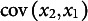 from Figure 2a are replaced with freely estimated parameters. The model-implied moments are again the marginal moments from Equations 10 and 12.

A main benefit of the stochastic approach is that missing data on the covariates can be handled directly with maximum likelihood assuming a missing at random mechanism because there are free parameters and distributional assumptions related to the covariates (Baraldi & Enders, 2010). In lavaan and M*plus*, this is specification is used whenever the mean or variance of a covariate is included in the code (or by using the fixed.x = FALSE option in lavaan). Similar to Section 4.4.1, the number of variables in the model is equal to *V* because all outcomes and covariates are considered part of the model.

#### 4.4.3 Conditional and fixed

A conditional specification aligns more closely with models from the regression or mixed effect tradition and the likelihood is conditioned on the covariates such that 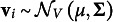 . In the conditional likelihood, the effects of covariates are removed from the model-implied moments correspond to the conditional moments in equations 9 and 11. The mean, variance, and covariance of covariates are fixed to sample statistics as in Section 4.4.1. Consequently, *P* is defined only as the number of focal outcome variables *T* rather than *V*. The corresponding model equations are,(15)

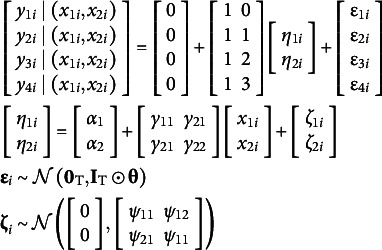



The path diagram corresponding to this specification is shown in Figure 2c. With a conditional and fixed specification, there are no distributional assumptions placed on the covariates, so missing covariates must be dealt with imputation or deletion (Sterba, 2014). The conditional and fixed specification is conceptually similar to the joint and fixed specification and the parameter estimates will closely correspond (and may be identical) even though there are different ramifications for defining SRMR.

Slope structures are not present in joint specifications but become relevant in conditional specifications (Muthén, 1984, pp. 49–50). The slope structure refers to possible pathways from the covariates in **x** to the outcomes **y** (possibly through latent variables in **η**) and corresponds to the covariance attributable to covariates (which is conditioned out). If the slope structure is saturated such that every covariate predicts every outcome (i.e., there are 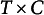 covariate paths in the model), then the observed covariance attributable to covariates will equal the model-implied covariance attributable to covariates. However, in the more common case where the slope structure is overidentified (i.e., there are fewer than 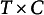 covariate paths), then there may be slope structure residuals in addition to the mean and covariance residuals for conditional specifications.

### 4.5 Covariate specification affects fit

Despite the conceptual similarity among specifications (especially without missing data where specifications yield identical parameter estimates), the choice of specification—whether made explicitly or implicitly by software—has implications for calculating SRMR because definitions of *P* are different. For the model in Figure 2, joint specifications are 6-dimensional, but the corresponding conditional specification is only 4-dimensional. Additionally, the conditional specification has different residuals because the variance attributable to covariates is removed whereas a joint specification yields marginal moments.

Section 5 provides different possible SRMR definitions that emerge from different covariate specifications and standardization methods. An empirical example and simulation follow in Section 6 to demonstrate complexities of defining model fit in complex models.

## 5 Defining SRMR with covariates

Sections 5.1 and 5.2 describe different SRMR definitions depending on the model specification. Key properties are summarized in Table 1. Section 5.3 discusses relevant properties to consider when choosing among different SRMR definitions for a model with covariates.

**Table 1:** Comparison of primary features of different possible SRMR definitions for models with covariates

Label	Moments	Covariate specification		Numerator	Denominator	lavaan	M*plus*
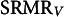	Marginal	Joint	Fixed or stochastic	δ_2_	V(V+3)/2	Default (srmr fit measure)	Covariate mean or variance in MODEL and INFORMATION = EXPECTED
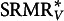	Marginal	Joint	Stochastic	δ_3_	V(V+3)/2	srmr_mplus fit measure	Covariate mean or variance in MODEL
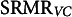	Marginal	Joint	Fixed	δ_2_	V(V+3)/2 – C(C+3)/2	NA	INFORMATION = EXPECTED
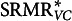	Marginal	Joint	Fixed	δ_3_	V(V+3)/2 – C(C+3)/2	NA	Default
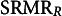	Conditional	Conditional	Fixed	δ_2_	T(T+3)/2	conditional.x=T, srmr fit measure	NA
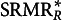	Conditional	Conditional	Fixed	δ_3_	T(T+3)/2	conditional.x=T, srmr_mplus fit measure	NA
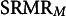	Marginal	Joint	Fixed or Stochastic	δ_2_	T(T+3)/2	NA	NA
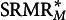	Marginal	Joint	Fixed or Stochastic	δ_3_	T(T+3)/3	NA	NA

*Note:* *V* = number of total variables in the model including covariates, *T* = number of focal outcome variables, *C* = number of covariates in the model. *V = T + C*. SRMR labels without a “*” are based on Bentler standardization that divides by sample standard deviations; SRMR labels with a “*” standardize using model-implied standard deviations for covariance and mean elements and sample standard deviation for the variance elements. M*plus* column refers to Version 8.10, lavaan column refers to version 0.6.17.

### 5.1 Conditional specification

The likelihood for a model with conditionally specified covariates is 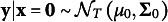 where 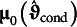 is the model-implied conditional means and 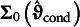 is the model-implied conditional covariance. The observed conditional means are then 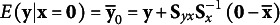 and the observed conditional covariance is 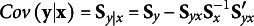 .

These conditional sample and model-implied moments can produce a residualized SRMR:(16)

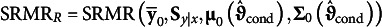




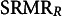
 provides an index of the standardized discrepancy between the sample and model-implied *conditional* means and *conditional* covariances of the *T* focal outcomes given all covariates are set to 0. This is most meaningful when covariates are centered or have natural zero points and when the interest is evaluating the discrepancy after removing variance explained by covariates.

Importantly, because 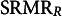 is conditional, changing the scaling of the covariates will change the value of 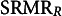 (e.g., centered versus uncentered covariates will have different values of 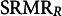 ). This can be useful if the fit at specific values of the covariates is desired because the scaling of the covariates can be adjusted so that the specific values of interest are set to 0.


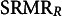
 in equation 16 uses Bentler standardization from equation 4, but it could use a mix of Bentler and Bollen standardization as in equation 5 such that 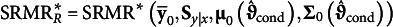 .

Regarding the slope structure, the observed covariance attributable to covariates is 
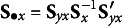
 and the model-implied covariance attributable to covariates is 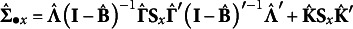 . The slope structure residuals are then equal to 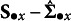 . The slope structure residuals are implicitly present in equation 16 because they are one source of misfit such that 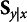 is based on removing all covariance attributable to covariates whereas 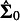 only removes covariance based on the specified, possibly overidentified slope structure. Separating the contribution of the slope structure residuals can help identify if misfit is potentially due to covariate-related covariance not being fully conditioned out.

### 5.2 Joint specification

Let 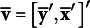 and 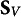 be the sample mean vector and covariance matrix for all *V* variables in a jointly specified model whose model-implied mean and covariance are 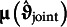 and 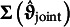 . These quantities could be used directly such that(17)

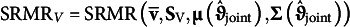




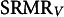
 uses the marginal model-implied and sample moments for all *V* variables in the model (i.e., the variance explained by covariates is not factored out). Essentially, 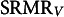 captures how well the model reproduces the means, variances, and covariances of focal outcomes *and* covariates simultaneously. 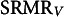 is applicable when covariates are treated as fixed or stochastic. Equation 17 uses Bentler standardization, but a corresponding 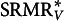 could also be defined by using the hybrid Bollen–Bentler standardization from equation 5.

Asparouhov and Muthén (2018) note that 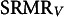 may be problematic with a fixed covariate specification because elements related to the covariates are included in the SRMR calculation, but they are constrained to sample values rather than being estimated. Therefore, these elements will not have misfit and cannot contribute to the SRMR numerator, but they will contribute to the denominator. Therefore, Asparouhov and Muthén (2018) describe a covariate adjustment for 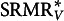 where(18)

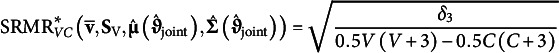



The denominator removes the 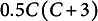 elements associated with the covariates whose model-implied values are constrained to sample values (which will necessarily have no misfit). Elements corresponding to covariates are factored out of the denominator to avoid artificially reducing the index by inflating the denominator. If *C* = 0, 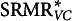 reduces to 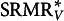 because none of the model-implied elements will be constrained to sample values. There is also a corresponding 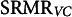 that corrects the denominator of 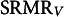 when Bentler standardization is used.

In M*plus* Version 8.10, specifying a model with stochastic covariates (e.g., by including the mean or variance of the covariate in the model) will result in 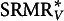 being reported in the output; the M*plus* default specifies fixed covariates and results in 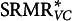 being reported in the output. The INFORMATION = EXPECTED option in M*plus* can yield 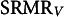 and 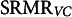 in the output but has ramifications beyond the calculation of SRMR. Specifically, it can affect the consistency of standard errors if missing data are present and not missing completely at random (Kenward & Molenberghs, 1998) and it can impact the effectiveness of robust estimators (Savalei, 2010). This may be particularly problematic for 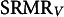 with a stochastic specification because a common motivation for this specification is accommodating covariates with missing values.

To extend the idea of 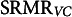 , the scope of SRMR can be refined further by subsetting the mean vector and covariance matrix to only include elements related to the *T* focal outcomes. This way, all means, variances, and covariances related to covariates are not counted in either the numerator or the denominator. Namely,(19)

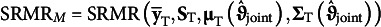

where 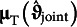 and 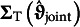 are the marginal mean vector and marginal covariance that only contain elements involving the *T* focal variables. After subsetting, 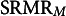 uses the same information regardless of whether covariates are fixed or stochastic. Because it uses marginal moments, 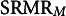 corresponds to the fit of unconditional growth model. 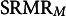 and 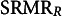 will be equivalent for mean-centered covariates that explain no variance because they will have the same dimension and the same moments. 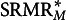 can be defined similarly but uses the hybrid Bollen-Bentler standardization (equivalence between 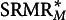 and 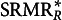 exists under the same conditions as equivalence of 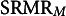 and 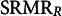 ).

### 5.3 Choosing among different definitions

Among the definitions in Sections 5.1 and 5.2, 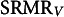 and 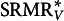 are the most susceptible to overly optimistic assessments of model fit when covariates are present, especially when a fixed specification is used for covariates. Both definitions use all variables—focal outcomes and covariates—in the numerator and denominator. The means and variances of covariates as well as covariances among covariates will fit exceedingly well because they are either explicitly fixed to sample statistics with a fixed specification (and will fit perfectly) or are the full information maximum likelihood estimates of sample statistics with a stochastic specification (Little & Rubin, 2020).


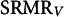
 and 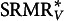 therefore capitalize on the perfect or near-perfect fit of the 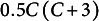 elements involving covariates and are susceptible to deflation because elements involving covariates contribute to the denominator but do not contribute or contribute very little to the numerator. Good fit can be achieved by simply adding many covariates into the model, which will increase the proportion of elements with 0 or near-zero residuals, which will attenuate 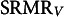 and 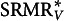 .

This mechanism motivates 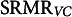 and 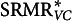 , which explicitly reduce the denominator by 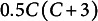 to account for elements that do not contribute to the numerator. This reduces—but may not entirely eliminate— 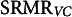 and 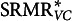 capitalizing on the presence of covariates and producing optimistic assessments of fit. In addition to the 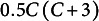 elements that will have perfect fit, there are 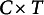 model-implied covariances that are partially informed by covariates. For instance, in Figure 2a, 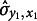 is implied (in part) by 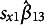 and 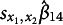 . The sample statistics will not have misfit and will limit the potential magnitude of misfit in 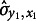 . Partial embedding of sample statistics throughout the model makes the effectiveness of a denominator correction for covariates uncertain because it is unclear how or whether to account for elements that have partial covariate information. As a result, 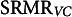 and 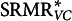 remain susceptible to some deflation when more covariates are added to the model. Nonetheless, the situation is somewhat ambiguous because the model’s ability to reproduce covariances between covariates and outcomes may be relevant because—even if these residuals are tempered—they are not guaranteed to be exactly zero.


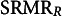
 , 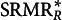 , 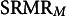 , and 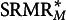 appear least susceptible to capitalizing on covariate information because they restrict focus solely to the *T* outcome measures. 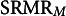 and 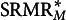 rely on the marginal mean and covariance, which preserves the interpretation as the fit of the unconditional model regardless of the number of covariates. This provides the most consistency by insulating the interpretation from the effect of covariates. However, as expanded upon in Sections 6.2 and 7, this may not necessarily be a positive characteristic. 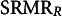 and 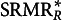 use the conditional mean and covariance, which is helpful to factor out explained variance. However, they are dependent on the scaling of the covariates and require a meaningful zero point for the covariates to have a meaningful interpretation. Essentially, 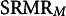 and 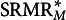 do not engage with the covariate information, which provides a constant interpretation. Conversely, 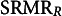 and 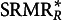 fully engage with covariate information, which results in a focused interpretation that is sensitive to changes in covariate scaling. The next section provides an empirical example to empirically demonstrate the differences between SRMR definitions.

## 6 Empirical example

### 6.1 Model description

To demonstrate how SRMR can be affected even for modest and routine models, this section uses a subset of the 1979 National Longitudinal Survey on Youth (NLSY). These data are publicly available and were retrieved from the companion site to the popular multilevel modeling textbook by Hox et al. (2018). The data feature Peabody Individual Assessment test reading scores (PIAT; Dunn & Markwardt, 1970) for 221 children. These data are wave-based such that each child’s PIAT score is measured four times in two-year intervals. This subsample has no missing values and each child completed all four waves.

For this example, a taxonomy of models were fit. Model 0 is an unconditional latent growth model. Model 1 includes mother’s age when the child was born (mom_age; *M* = 25.59, *SD* = 1.87, range = 21–29) as a time-invariant covariate of the initial status and linear change growth factors. Model 2 adds a second time-invariant covariate, cognitive support provided at home (cog_home; *M* = 9.10, *SD* = 2.45, range = 3–14). Both covariates were grand-mean centered to preserve the interpretation of the growth factor means. The models were fit with maximum likelihood estimation in lavaan Version 0.6.17 (Rosseel, 2012) or in M*plus* Version 8.10 (Muthén & Muthén, 1998–2024). Because there was no missing data, parameter estimates are identical across specifications and programs.

The model 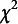 and degrees of freedom are reported in Table 2 along with the estimated parameters for each model and SRMR values from each definition in Section 5. Parameter estimates are identical across all specifications. The data and code for this example are provided on an Open Science Framework page associated with this paper, (https://osf.io/sxp4g). Software options yielding different SRMR definitions are provided in Table 1. 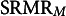 and 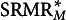 are not currently available in either software and were computed manually.

**Table 2:** Parameter estimates from three latent growth models fit to the empirical reading assessment data

	Parameter		Model 0		Model 1		Model 2	
	Joint	Conditional	Est	SE	Est	SE	Est	SE
Mean structure								
Initial status	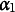		2.83	0.06	2.83	0.06	2.83	0.06
Linear change	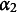		1.05	0.02	1.05	0.02	1.05	0.02
Initial status on mom_age	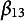	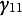			0.09	0.03	0.08	0.03
Linear change on mom_age	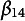	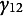			0.03	0.01	0.03	0.01
Initial status on cog_home	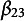	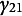					0.03	0.02
Linear change on cog_home	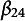	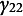					0.01	0.01
Covariance structure								
Var(initial status)	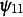	0.48	0.08	0.45	0.08		0.44	0.08
Var(linear change)	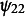	0.04	0.01	0.04	0.01		0.04	0.01
Cov(initial, linear)	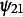	0.07	0.03	0.06	0.03		0.06	0.02
Wave 1 Res. Var	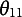	0.55	0.07	0.55	0.07		0.54	0.07
Wave 2 Res. Var	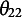	0.26	0.03	0.26	0.03		0.26	0.03
Wave 3 Res. Var	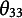	0.21	0.03	0.21	0.03		0.21	0.03
Wave 4 Res. Var	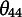	0.36	0.06	0.36	0.06		0.35	0.06
Model fit								
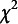		124.11		124.38			125.32	
Df		5		7			9	
Bentler standardized								
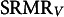			.163		.136		.118	
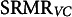			.163		.144		.130	
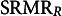			.163		.165		.163	
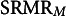			.163		.163		.163	
Bollen–Bentler standardized								
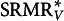			.112		.093		.080	
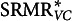			.112		.098		.089	
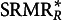			.112		.113		.112	
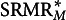			.112		.112		.112	

*Note:* Est = parameter estimate, SE = standard error, df = degrees of freedom, Res. Var. = residual variance, Cov = covariance, Var = variance

### 6.2 Model results

All three models have identical model 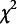 statistics for all specifications. The model 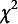 values are larger than the critical value for any conventional significance level given the model degrees of freedom, indicating that the models do not recreate the sample moments exactly. Because the model 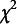 test can be seen as a severe test for data-model fit (Mayo, 2018), SRMR can help quantify the magnitude of the model residuals to help contextualize the practical amount of data-model misfit. Because there are no covariates in Model 0, the Bentler-standardized indices 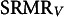 , 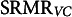 , 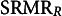 , and 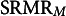 are identical. Similarly, the hybrid Bollen–Bentler standardized indices 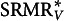 , 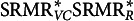 , and 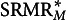 are also equal to each other, but they are not equal to the Bentler-standardized SRMRs because the variance structure is not saturated and 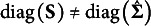 .

As covariates are added, 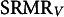 , 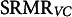 , 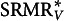 , and 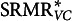 systematically decrease regardless of whether the covariates improve the model. That is, despite home_cog having no effect on initial status ( 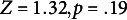 ) or linear change ( 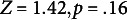 ), all four of these SRMR definitions suggest improvement between Model 1 and Model 2. This behavior stems from relying on *V*-dimensional moments, which rewards reproduction of covariate elements.

To demonstrate, the Model 2 Bentler-standardized residual mean vector and residual covariance matrix for a joint covariate specification are

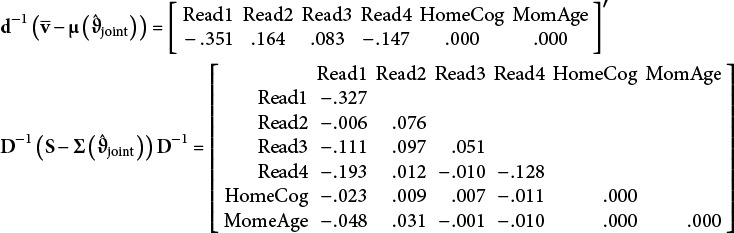



Notably, there are 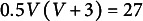 unique elements across the mean vector and covariance matrix. The sum of squared standardized residuals is 0.374 (the numerator of 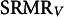 ) but notice that two rightmost elements in the mean residual vector and three elements in the rightmost lower triangle of the covariance residual matrix are necessarily zero because they only involve covariate information. Because there are no missing data, the model-implied elements are identical to the sample statistics regardless of whether a fixed or stochastic specification is used. This produces zero residuals for these elements, so they do not contribute to the numerator but they do add to the denominator, resulting in 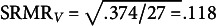 for Model 2.


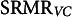
 subtracts 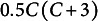 from the denominator to address deterministic zeroes. In Model 2, *C* = 2 so the denominator is lowered by 5 to account for values that are necessarily 0 (i.e., 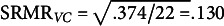 ). 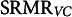 continues to count the 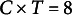 elements that are partially based on the covariates (the 8 non-zero values in the last two rows of the residual covariance matrix above). These elements represent the discrepancy of the model-implied and observed covariances between the repeated measures and the covariates.

One perspective of these elements is that these elements should count towards SRMR because reproducing the covariance between the outcome and covariates is relevant information to consider. From this perspective, reproduction is the main interest and the decrease in 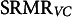 is warranted because it indicates that the model is adequately reproducing the covariances between repeated measures and covariates.

An alternative perspective is that the model should not be rewarded for small residuals that are partially composed of sample statistics. Although these elements are not deterministically zero, their magnitude is moderated—for instance, these elements represent 8 of the 11 smallest non-zero residuals in the model. From this perspective, counting elements that are partially dependent on covariate sample statistics artificially deflates 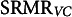 because there is not a substantive interest in reproducing these covariance elements and counting them drives down the 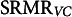 without providing substantively useful information. From this perspective, determining whether the covariates improve the model is the main interest and the decrease in 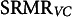 is less warranted because it does not speak to whether the covariates are useful or whether the model-implied covariances of the repeated measures more closely reproduce the observed covariances after covariates are included. This is particularly prudent for researchers intending to use 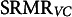 for model comparisons or fit index difference evaluation because there is a distinction between a model reproducing the covariances between outcomes and covariates and the covariates explaining variance in the outcome.

Regarding other definitions, 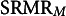 and 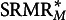 do not change across the three models. This stability is due to these definitions marginalizing over the covariates, so 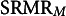 and 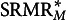 will be constant whether covariates are present or not. For these data, the Bentler-standardized residual mean vector and residual covariance matrix used for 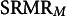 and 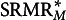 only include the *T*-dimensional elements related to the four repeated measures:

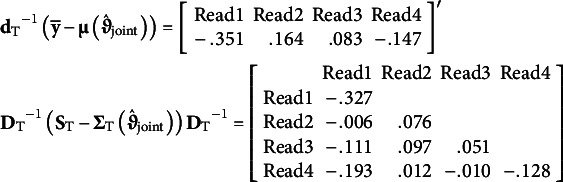



where 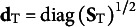 and 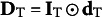 . There are only, 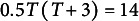 unique elements, resulting in a sum of squared residuals of .369 and 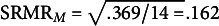 . The calculation involves no covariate information and focuses purely on fit of repeated measure elements.


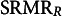
 and 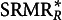 happen to mirror 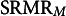 and 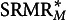 , respectively, in this example but this will not be the case generally. Covariates in the example are grand-mean centered and explain little variance, so the marginal and conditional definitions converge. But if Model 2 is refit using uncentered covariates, 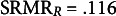 and 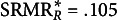 and conditional moments no longer correspond to the marginal moments because SRMR is now conditional on the covariates equaling 0 on their original scales. Similarly, if the interest was fit of Model 2 for people simultaneously at the maximum values of the two covariates, the model could be refit centering the covariates around their maximum values. This yields 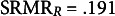 and 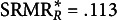 suggesting that the model fits less well for people at the upper extreme of the covariates. Other SRMR definitions are based on marginal moments and are unaffected by covariate centering.

This reinforces the point made earlier: although 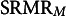 and 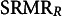 are both resistant to artificial deflation when adding covariates and can converge in some cases. However, resistance is gained through two opposing mechanisms. 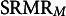 is not deflated by covariates because its interpretation is insensitive and oblivious to the presence of covariates. Conversely, 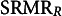 is not deflated by covariates because its interpretation is entirely dependent on the covariate information and its value changes within the same model as a function of covariate scaling.

### 6.3 Expanding Figure 1

**Figure 3:**
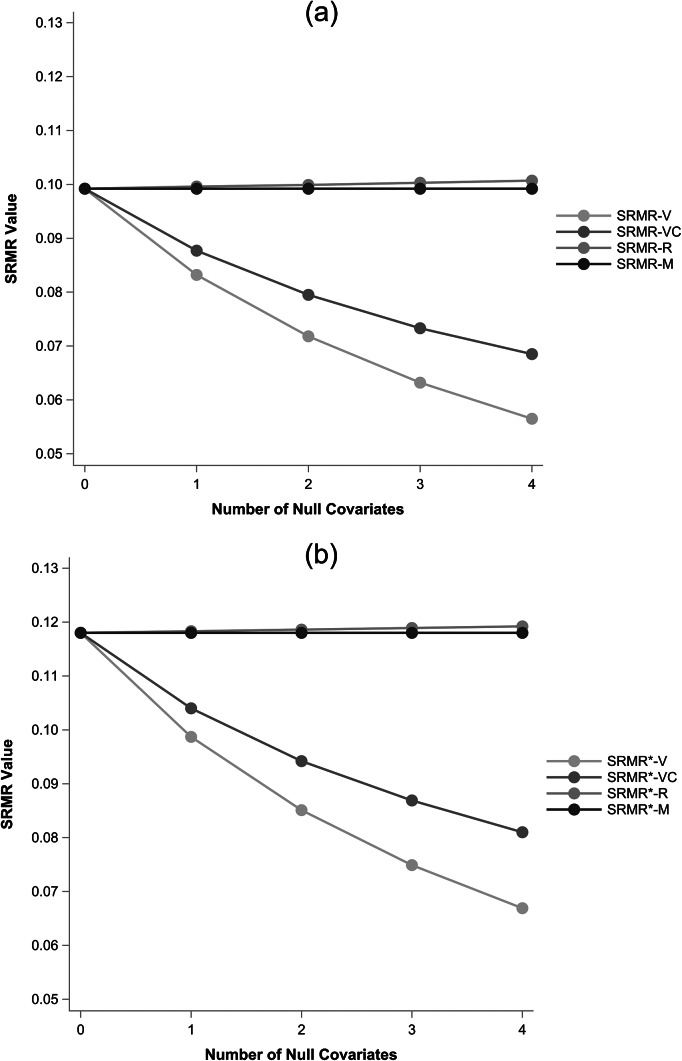
Simulation results showing average SRMR value across replications as the number of null covariates increases. Panel (a) shows the Bentler-standardized SRMR definitions and panel (b) shows the Bollen-Bentler standardized SRMR definitions. Patterns in simulated data match those in the empirical example where 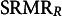 and 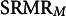 are stable and unaffected when null covariates are added whereas 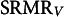 decreases sharply and 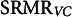 decreases but more moderately.

Data in Section 6.2 were empirical and the truth is not concretely known. All SRMR definitions from Section 5 were therefore applied to the same simulated data from Section 2 that were used to create Figure 1. The results are shown in Figure 3 where Panel A shows Bentler standardized definitions and Panel B shows Bollen-Bentler standardized definitions.

Results in Figure 3 mirror those in the empirical example. Namely, when truly null covariates are added to the model, 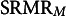 maintains a consistent value, 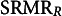 is mostly consistent with small variation due to variance explained by random chance, 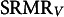 sharply decreases as more null covariates are added because a larger proportion of elements are deterministic zeroes, and 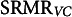 decreases but less sharply because it filters out the deterministic zeroes but still includes elements that are partially based on sample statistics. Patterns are the same for either standardization method.

## 7 Discussion

The traditional definition of SRMR is appropriate for covariance structure models, but many structural equation model applications include additional features, which can alter the appropriate definition of SRMR. The current paper focused on the context of models with covariates. Despite mainstream software reporting SRMR values for models featuring covariates, there has been little formal methodological work exploring how to suitably extend SRMR when covariates are present.

The primary finding in this paper was that some SRMR definitions were susceptible to being systematically deflated if covariates are present 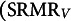 , 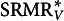 , 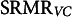 , and 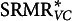 ). Other definition were less susceptible 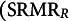 , 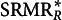 , 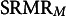 , and 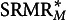 ), but had properties and interpretational caveats that may be undesirable. This is primarily due to joint covariate specifications, which can complicate counting which variables are “in the model” and which model residuals should contribute to the numerator and denominator of SRMR.

Consequently, this paper does not definitively solve the issue of properly defining SRMR with covariates and it may raise more questions than answers. It also only considered the situation with continuous outcomes and did not explore contexts where outcomes are discrete (see Section 2.7 of Asparouhov & Muthén, 2018 for SRMR considerations with discrete outcomes). Nonetheless, the hope is that this paper at least raises awareness of these potential issues and encourages more thoughtful consideration of how to interpret SRMR when models feature covariates.

Regarding specific limitations of definitions that did not systematically decrease when covariates were included, 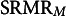 and 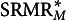 mirror evaluating fit with no covariates because covariates are marginalized out, which does not differentiate between explained and unexplained variance. 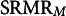 and 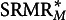 are essentially oblivious to covariates because the variance is pushed around to different sources, but the marginal amount of variance is unchanged. Essentially, 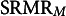 is not deflated with covariates because it simply is not sensitive to covariates. 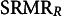 and 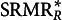 are conditional, which seems more useful because the variance explained by covariates is removed. However, its interpretation depends on how covariates are scaled.

In essence, a meaningful SRMR with covariates may require a better developed sense of what the model-implied moments should reproduce. This is closely related to the ambiguity when interpreting 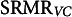 and 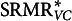 where it is unclear whether elements corresponding to covariances between outcomes and covariates are part of the model and whether reducing them meaningfully corresponds to what fit should be capturing.

Moreover, it is unclear which values of any SRMR definitions for covariates indicate acceptable approximate fit. The traditional guideline from Hu and Bentler (1999) is that SRMR < .08 indicates acceptable fit. However, this guideline is known not to generalize well beyond the confirmatory factor models from which it was derived (e.g., Fan & Sivo, 2007; Hancock & Mueller, 2011; McNeish & Wolf, 2024). Models with covariates differ in meaningful ways from Hu and Bentler’s simulation, so it is unclear which SRMR values indicate substantively important misfit when covariates are present. This is especially true for models with an overidentified mean structure because the mean and covariance structure may be weighed differently.

This also raises questions about whether combining mean structure residuals and covariance structure residuals into a single index is meaningful. As noted earlier, mean residuals and covariance residuals can be split with separate SRMR values for each submodel to make the index more interpretable and to better identify the location of misfit. Nonetheless, the same issues discussed in this paper are relevant even if using separate SRMRs for the mean and covariance structure because there are still ambiguities about which elements should and should not be counted in each (though the mean structure is less opaque than the covariance structure).

Of course, SRMR may simply have too many interpretational challenges to productively assess approximate fit for models with covariates. Whereas lack of dependence on *T*_ML_ can be a helpful property of SRMR in covariance structure models, SRMR’s reliance on model residuals may convolute calculation of SRMR in situations where there is ambiguity regarding which variables and corresponding residual elements should be counted as part of “the model”. If a concise summary of residuals with SRMR may be too difficult, local fit may offer another option for those looking to evaluate fit of models with covariates.

Broadly, local fit refers to evaluating some smaller portion of the overall model (Thoemmes et al., 2018). This can include structural versus measurement portions (Anderson & Gerbing, 1988; Zhang & Hao, 2024) or can be as narrow as elementwise inspection of each individual residual element to identify elements that were not closely reproduced by the model (McDonald & Ho, 2002; West et al., 2023). Elementwise local fit is essentially the most extreme version of splitting SRMR into subcomponents and can help locate areas of local strain and to ensure that misfit is not attributable to a few outlying elements that are poorly reproduced (Appelbaum et al., 2018; Kline, 2023).

Elementwise local fit is commonly recommended as a supplement to global fit metrics like SRMR. However, reviews of empirical studies suggest that few studies report or examine model residuals and elementwise local fit and instead rely on global summary measures like SRMR (Ropovik, 2015; Zhang et al., 2021). As models become more complex, it may be more straightforward to simply look at each residual in isolation rather than debate merits of different possible aggregated summaries of the residuals.

Similar to global fit, elementwise local fit can be exact or approximate. In exact approaches, inferential tests are built to assess whether the individual residual element is equal to 0 (Maydeu-Olivares, 2017; Ogasawara, 2001). With approximate local fit, the intent is to identify whether the amount of misfit for an individual element is acceptably small. Typical recommendations for elementwise local approximate fit are that standardized residuals are between [–0.10, 0.10] (Hu & Bentler, 1995; Goodboy & Kline, 2017; Schreiber, 2008). However, this recommendation is motivated by factor analysis and may not apply to models with overidentified mean structures where residuals are not bounded. Additional work that refines understanding of elementwise local fit in models that extend beyond factor analysis would be beneficial.

In sum, hopefully this paper has illuminated potential issues and open problems when extending approximate fit indices like SRMR that were originally developed for the narrower context of covariance structure models. Even though there are few definitive conclusions, hopefully researchers will have greater appreciation for nuance required when using any SRMR definition to understand the data-model fit when covariates are present.

## Footnotes

1 Note that M*plus* uses a slightly different definition of SRMR than defined by Bentler (1995); see Equation 129 in Muthen (2004, p. 23). This is discussed in more detail in Section 3.4.

2 If covariates have known imperfect reliability, residual variances could be fixed to a non-zero value that implies a particular reliability (Bollen, 1989, p. 312; Cole & Preacher, 2014).
